# Regulatory mechanisms of deer antler extracellular vesicles in multilevel tissue repair: a state-of-the-art review

**DOI:** 10.3389/fphar.2026.1758263

**Published:** 2026-02-19

**Authors:** Lei Yuan, Bowen Deng, Fengrui Zhang, Deyou Wang, Haiyan Chen

**Affiliations:** Changchun Sci-Tech University, Changchun, Jilin, China

**Keywords:** deer antler, extracellular vesicles, immune microenvironment, regenerative medicine, signalling pathways, tissue repair

## Abstract

Extracellular vesicles (EVs) are defined as key nanoscale messengers that mediate intercellular communication, demonstrating immense potential in tissue repair and regenerative medicine. As the only organ in mammals capable of complete, cyclical regeneration, deer antlers provide EVs with exceptional regenerative bioactivity. This paper systematically reviews and prospectively discusses the research field of deer antler-derived EVs. We first outline their isolation strategies and characteristic functional subtypes, then focus on elucidating their multi-level molecular mechanisms driving tissue repair: at the cellular level, they directly regulate stem cell proliferation and lineage differentiation; at the microenvironmental level, they effectively remodel the immune ecology of injured areas by reprogramming immune cells and coordinating cytokine networks, thereby creating favorable conditions for regeneration. At the molecular level, they precisely regulate core signaling pathways, including the Wnt/β-catenin, NF-κB, miR-21-5p/STAT3, and TGF-β pathways. Finally, this paper prospectively explores cutting-edge developments in the field, including enhancing vesicle targeting and drug-loading capacity through engineering strategies, constructing controlled-release delivery systems based on smart materials, and developing precision therapies tailored to specific pathological microenvironments. This review aims to elucidate the biomedical potential of deer antler extracellular vesicles as regenerative nanomedicines for promoting tissue repair.

## 1 Introduction

In the field of regenerative medicine, extracellular vesicles (EVs) derived from stem cells have emerged as pivotal mediators for intercellular communication and tissue repair regulation due to their core function of targeted delivery of bioactive molecules (Wu et al., 2025b). Among numerous stem cell subtypes, deer antler stem cells stand as the only adult stem cells capable of driving lifelong periodic complete regeneration of mammalian organs (Wang et al., 2025a). Their unique biological characteristics offer irreplaceable research value for developing highly effective regenerative repair-oriented EV products.

Compared to traditional adult stem cells, antler stem cells possess highly distinctive core characteristics. They exhibit rapid proliferation capacity. During the antler regeneration cycle, they can rapidly proliferate and differentiate into large numbers of functional cells, providing an abundant cellular source for organ regeneration (Li et al., 2023). The antler regeneration process is essentially a continuous cycle of trauma repair and tissue reconstruction. Antler stem cells exhibit exceptional trauma healing regulation capabilities, efficiently recruiting repair-related cells through paracrine effects, suppressing inflammatory responses, and accelerating wound healing (Guo et al., 2025b). Furthermore, the unique capacity for complete organ regeneration in deer antler stem cells effectively coordinates the synergistic differentiation and orderly reconstruction of multiple tissues—including bone, cartilage, blood vessels, and nerves—ultimately enabling the periodic regeneration of the entire antler organ (Dong et al., 2019).

The unique biological characteristics of deer antler stem cells confer superior properties to their derived EVs. The rapid proliferation of stem cells enriches their EVs with microRNAs regulating cell cycle progression and growth factors promoting proliferation. These molecular cargoes directly accelerate target cell cycle processes, enhancing cellular proliferation efficiency during tissue repair (Yang et al., 2020b). Their wound healing regulatory properties confer a high abundance of anti-inflammatory factors and chemotactic molecules, enabling more efficient suppression of inflammatory responses, aggregation of repair cells, and enhanced wound repair capacity (Dong and Coates, 2021). The capacity for complete organ regeneration allows their EVs to carry key growth factors for osteochondral differentiation and signaling proteins regulating tissue remodeling pathways, thereby coordinating multi-tissue synergistic differentiation and providing core support for complex tissue and organ repair (Li et al., 2025a).

Therefore, this paper systematically reviews the current research status of EVs derived from deer antler velvet, elucidates EVs’ isolation methods, and evaluates the characterization of deer antler-specific EV subtypes. It reveals the complex mechanisms by which these EVs regulate tissue repair, with a focus on immune microenvironment remodeling and signaling pathway modulation. Finally, this review explores emerging frontiers, including EVs engineering for enhanced targeting, the development of advanced delivery systems, and their potential in disease-specific therapies, providing a theoretical foundation for regenerative medicine applications based on antler stem cell-derived EVs.

## 2 Structural characteristics of EVs

EVs are membrane-bound vesicles secreted by nearly all cell types, containing cell-derived molecules such as lipids, proteins, nucleic acids, and metabolites (Cocozza et al., 2020; Sun et al., 2019). Based on origin and size, EVs are classified into three types: exosomes, microvesicles (MVs), and apoptotic bodies (ABs). Exosomes originate from the endosomal system, are produced via multivesicular secretion, have diameters of approximately 30–150 nm, densities of about 1.13–1.19 g/mL, and surfaces enriched with markers such as CD63, CD81, and TSG101. MVs originate from budding of the cell membrane, with diameters ranging from approximately 100–1,000 nm, and densities of about 1.05–1.10 g/mL. Their surfaces are rich in markers such as CD40 and integrins. ABs originate from the fragmentation of apoptotic cells, with diameters ranging from approximately 1,000–5,000 nm and densities of about 1.16–1.28 g/mL. Their surfaces are rich in markers such as phosphatidylserine and histones (Wang et al., 2024; Ohno et al., 2016; Nieuwland et al., 2018).

## 3 Isolation of EVs

The extraction of EVs is a fundamental step for their research and application (Nasiri Kenari et al., 2020). Animal-derived EVs are typically extracted from bodily fluids and subsequently separated from protein and lipoprotein contaminants (Coumans et al., 2017). The challenges in isolating and purifying EVs lie in the viscosity of bodily fluids and the presence of non-EVs proteins and lipid particles in serum (Brennan et al., 2020). For EVs derived from deer antler velvet, unique tissue-specific technical difficulties further complicate the isolation process, primarily related to the handling of calcified matrix and cartilage digestion. Antlers constitute a specialized hierarchical tissue composed of cartilage, pre-mineralized tissue, and calcified tissue. The dense calcified matrix and resilient cartilage not only impede the efficient release of EVs from the tissue but also readily release non-targeted impurities during sample processing, interfering with subsequent isolation steps (Chen et al., 2024). Specifically, the calcified matrix of antlers is rich in hydroxyapatite, exhibiting high density and hardness. Improper handling during homogenization and centrifugation can cause mechanical damage to EVs, while released calcium ions may compromise their stability (Gonzalez et al., 2025). Concurrently, the cartilaginous tissue of antlers contains abundant collagen fibers and proteoglycans, forming a dense extracellular matrix network that encapsulates EVs (Zuo et al., 2024). This hinders their diffusion into the extraction solution. Overdigestion of cartilage may disrupt the membrane structure of EVs, while underdigestion results in low EV yield. For calcified matrices, chelating agents like EDTA or citrate are commonly employed to specifically bind calcium ions in hydroxyapatite, enabling gentle decalcification without damaging EV membranes (Shapiro and Landis, 2024; Azoidis et al., 2018). Alternatively, low-temperature mechanical homogenization combined with gradient sonication can break down calcified matrices while minimizing mechanical shear forces on EVs. For cartilage digestion, a composite enzyme mixture comprising type II collagenase, hyaluronidase, and dispersin specifically degrades collagen fibers and proteoglycans within the extracellular matrix. Adding protease inhibitors ensures complete EVs release while preventing excessive digestion of EVs membranes (Catalano and O’Driscoll, 2020).

**FIGURE 1:**
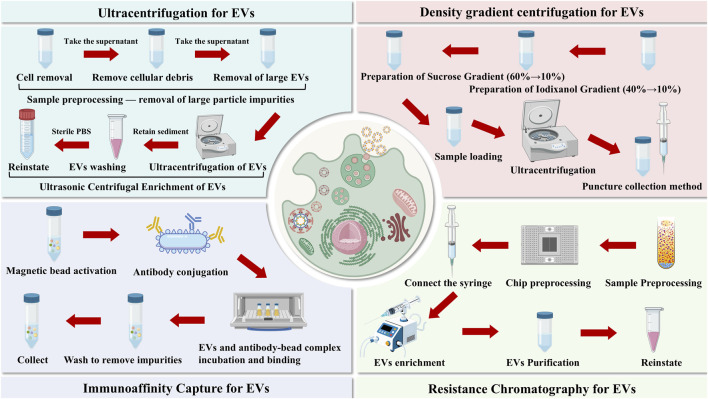
Different methods for extracting and isolating extracellular vesicles.

Based on these targeted pretreatments, further separation of deer antler-derived EVs was achieved through centrifugation, filtration, chromatography, or other methods, leveraging differences in size, density, sedimentation coefficient, and osmotic pressure between EVs and residual contaminants (cellular debris, heterologous proteins, nucleic acids, etc.) (Figure 1) (Takahashi and Takakura, 2023). Furthermore, techniques targeting specific surface markers on EVs (CD9, CD63, CD81, etc.) enable precise EV capture via antigen-antibody binding, ligand-receptor interactions, or receptor-mediated capture, while excluding extraneous interference (Vader et al., 2016). Currently, commonly used methods for EVs extraction include ultracentrifugation, density gradient centrifugation, size exclusion chromatography, tangential flow filtration, immunoaffinity capture, and microfluidic chip technology (Table 1). Among these, ultracentrifugation is widely regarded as the gold standard in this field (Alexandre et al., 2024). When combined with the aforementioned pretreatment strategies, it significantly enhances the yield and purity of deer antler-derived EVs.

**TABLE 1:** Systematic evaluation of EVs isolation methods.

Extraction method	Advantages	Limitations	Critical impact on functionality
Ultracentrifugation (UC)	Highly reproducible, compatible with various samples, free from chemical interference, high recovery rate, and operation can be optimized.	Equipment limitations, time-consuming process, limited purity, and potential damage to EVs.	High recovery rate and no chemical interference ensure consistent functional detection results with good reproducibility, but limited purity may cause non-EVs component interference, and potential EVs damage may underestimate actual functional activity.
Density gradient centrifugation (DGC)	High purity, excellent subtype separation capability, good activity retention, and controllable impurities.	Complex procedures, longer processing times, higher costs, and lower recovery rates.	High purity and superior subtype separation improve the accuracy of functional positioning for specific subtypes, while low recovery rate may miss rare functional subtypes and longer processing times may degrade labile molecules, affecting result reliability.
Immunoaffinity capture method	Highly specific, no centrifugation required, precise subtype enrichment, flexible operation.	Extremely high cost, low yield, risk of antibody residues, and high dependence on sample quality.	High specificity enables targeted research on marker-positive EVs to reflect target population functions, but antibody residues may distort results, low yield limits large-scale verification, and high sample quality dependence easily leads to functional misjudgment.
Microfluidic chip method	Micro-sample compatibility is strong, highly efficient, and rapid, with minimal time consumption and high integration. It simplifies workflows while maintaining good activity preservation.	Equipment and production costs are extremely high, scalability is extremely poor, sample preprocessing is highly demanding, maintenance costs are high, and stability is poor.	Strong micro-sample compatibility and rapid efficiency suit scarce samples and dynamic functional monitoring, but poor stability reduces result reproducibility, harsh preprocessing may alter EVs characteristics, and poor scalability limits the representativeness and universality of functional conclusions.

### 3.1 Ultracentrifugation

The ultracentrifugation method combines microcentrifugation with ultracentrifugation. Through sequential centrifugation, it progressively removes cell debris and large vesicles, ultimately precipitating EVs at the final centrifugal speed (Gupta et al., 2018; Jakl et al., 2023). This separation method relies on particle size and density differences (Ansari et al., 2024), can process samples from various animal sources, including cell culture supernatants, serum, and plasma (Lee et al., 2019), and preserves different EV subtypes. Centrifugation speed, wash frequency, duration, and purification factor must be selected based on the sample origin. These parameters not only influence the quantity and quality of isolated EVs (Zhao et al., 2022), but variations significantly alter EVs’ composition, biological activity, and the reproducibility of research outcomes. Excessively high centrifugation speeds may cause co-precipitation of protein aggregates with similar densities. Inadequate washing leaves residual serum proteins like albumin and immunoglobulins, while excessive washing may strip soluble proteins bound to EVs’ surfaces (Sorkin et al., 2017). Inconsistent centrifugation times fluctuate the recovery rate of small-sized EVs, altering the proportion of miRNA and lncRNA within the EV population. Concurrently, mechanical stress from high centrifugal forces may disrupt EVs’ lipid bilayers and alter membrane protein conformation, diminishing the chondrocyte proliferation-promoting activity of deer antler EVs (Morshed et al., 2020). Furthermore, the centrifuge’s maximum speed and sample viscosity determine the K-factor and separation efficiency, respectively (Bharti et al., 2025). The current lack of standardized parameters across studies leads to inconsistent reports on deer antler EVs particle size distribution, protein biomarker expression levels, and biological activity, severely compromising the comparability.

### 3.2 Density gradient centrifugation

Density gradient centrifugation, similar to ultracentrifugation, relies on density differences between biological particles to achieve precise separation. However, this method employs sucrose or iodohydroxybenzyl alcohol to form a continuous density gradient zone within the density gradient medium. Following ultracentrifugation, EVs are enriched within their specific density range, separating from impurities of differing densities such as protein aggregates, microvesicles, and apoptotic bodies (Zhang et al., 2014). Nevertheless, variations in gradient medium selection, concentration gradient design, and operational details can significantly impact the composition, biological activity, and reproducibility of EVs research outcomes. Sucrose gradients may cause partial adsorption of sucrose molecules onto EVs due to osmotic pressure changes. While hydroxybenzyl iodide offers higher separation precision, it may slightly damage the lipid bilayer, leading to endogenous lipid leakage or exogenous impurity infiltration—both of which alter EVs’ lipid composition. Furthermore, different media and gradient settings introduce variable residual impurities (Yuana et al., 2014). These impurities may interact synergistically or antagonistically with EVs, interfering with bioactivity assessments and reducing inter-laboratory reproducibility of research outcomes. Additionally, inconsistencies in operational details—such as gradient preparation time and centrifugation temperature—further exacerbate variations in EVs enrichment purity and bioactivity preservation, making it difficult to compare results across similar studies.

### 3.3 Size exclusion chromatography

Size exclusion chromatography (SEC) is a liquid chromatography technique that separates molecules based on size differences. It efficiently isolates deer antler EVs while removing impurities such as free proteins and nucleic acids. With its gentle operation and ability to preserve the natural structure and activity of EVs, SEC is a commonly used method for EVs separation, purification, and quality control analysis (Böing et al., 2014; Benayas et al., 2023). SEC columns are packed with porous microspheres (cross-linked dextran, agarose, polyacrylamide, etc.), featuring internal mesh structures with varying pore sizes. When the sample solution flows through the column, molecules larger than the pore size cannot enter the microsphere interior and rapidly elute along the external water volume between microspheres, resulting in short retention times. Small molecules smaller than the pore size can freely diffuse into the microsphere interior, traversing a longer path and exhibiting longer retention times (Monguió-Tortajada et al., 2019). By collecting fractions eluted at different times, deer antler EVs can be separated from impurities, while simultaneously enabling analysis of EVs’ particle size distribution and concentration. Notably, variations in SEC method parameters across studies can introduce research bias: different filler types and elution flow rates affect separation efficiency. Low flow rates reduce non-specific binding of EVs to the filler but increase EV aggregation, altering proteomic detection results (Liu et al., 2020). Differences in pH and ionic strength of the elution buffer affect the stability of EVs surface charges, thereby altering their binding capacity to target cells (Blans et al., 2017). SEC inherently struggles to efficiently separate EV subpopulations with similar particle sizes and exhibits relatively low throughput, making it more suitable for small-scale EVs and quality control. Furthermore, the lack of standardized column specifications and elution conditions across studies hinders the comparability of purity testing and activity evaluation results for EV isolates from the same deer antler source, limiting their application in large-scale comparative research.

### 3.4 Tangential flow filtration

Tangential flow filtration (TFF) is a size-exclusion-based membrane separation technology. The sample solution flows at high speed parallel to the membrane surface. The permeate passes through the membrane pores under transmembrane pressure, while the retentate flows along the membrane surface. Separation is achieved based on the membrane’s molecular weight cutoff (MWCO) or pore size (Kim et al., 2021). Typically, ultrafiltration membranes with an MWCO of 100–500 kDa can retain EVs with diameters of 30–200 nm, while small-molecule proteins, inorganic salts, metabolites, and other components in the culture medium are discharged with the permeate. The shear force generated by parallel flow rinses the membrane surface, preventing the formation of a filter cake layer composed of cellular debris, collagen, and other impurities. This maintains stable filtration efficiency while minimizing structural damage to EVs caused by compression or blockage, thereby maximizing the preservation of their biological activity (Visan et al., 2022). Additionally, TFF enables simultaneous concentration and washing, eliminating the need for multiple centrifugation steps and simplifying the workflow. It is often combined with SEC to further remove residual protein aggregates, yielding highly purified EVs (Busatto et al., 2018). The impact of methodological differences on EVs research primarily manifests in membrane parameters and operational conditions. Selecting different MWCO membranes results in varying EV subpopulations retained; for instance, 500 kDa membranes capture more large-sized microvesicles, increasing the proportion of lipid-rich EV fractions (Kawai-Harada et al., 2024). Differences in cross-flow pressure settings significantly impact EVs’ activity. Excessive pressure causes EVs deformation and disruption of membrane surface antigen structures, reducing their ability to specifically recognize target cells (Kim et al., 2025b). Furthermore, inconsistent settings for flow rate and filtration cycles across studies cause fluctuations in EVs recovery rates and residual impurities, thereby compromising the reproducibility of subsequent bioactivity assays. For instance, evaluations of antler velvet EV-mediated tissue repair often exhibit result discrepancies due to varying operational parameters.

### 3.5 Immunoaffinity capture method

This method utilizes antigen-antibody specific binding to isolate EVs without requiring ultracentrifugation, enabling precise capture of target EVs. It is particularly suitable for highly specific enrichment of deer antler EVs (Sidhom et al., 2020). Common surface markers expressed on EVs include CD9, CD63, and CD81, alongside tissue-specific markers such as PRG4 and IGF-1 receptor in antler EVs. By immobilizing specific antibodies onto a carrier, these antibodies can specifically recognize and bind to exosomal surface markers after incubation with the sample. Unbound impurities are removed through washing, ultimately eluting highly purified EVs (Hofmann et al., 2020). Methodological variations significantly impact research consistency. The choice of capture antigen influences the enriched EVs subpopulations: CD63-positive EVs are rich in immunoregulatory proteins, while PRG4-positive EVs favor components associated with cartilage repair. Without a clear justification for antigen selection, compositional analyses of antler EVs cannot be directly compared across studies (Fernandes et al., 2025). Antibody immobilization methods and elution conditions also impact EV activity. Acidic eluents efficiently dissociate antigen-antibody complexes but may disrupt EVs’ membrane structure and internal bioactive molecules. Physically adsorbed antibodies can detach and contaminate EV samples, interfering with EV-target cell interactions. Furthermore, inconsistent settings for antibody concentration and incubation time across studies cause fluctuations in EVs capture efficiency and purity, reducing the reproducibility of reported biological activity results (Zhang et al., 2021a). Although immunocapture facilitates the isolation of specific exosome subpopulations from complex mixtures and may distinguish different EV types, the limitation that capture antigens can only target surface-expressed markers—not intracellular antigens—combined with methodological variations, impacts the reliability of research outcomes.

### 3.6 Microfluidic chip method

By precisely controlling fluid dynamics, electrical interactions, or biomolecular interactions through microchannel structures in microfluidic chips, EVs can be separated from impurities (Zhang et al., 2025). The primary mechanisms employed for separating deer antler EVs include size-exclusion mechanisms and composite mechanisms. Size-exclusion separation utilizes nanoporous membranes or microcolumn arrays to trap deer antler EV particles, filtering out larger tissue debris and protein aggregates. Composite mechanisms integrate deterministic lateral displacement and dielectrophoresis, achieving precise separation by exploiting size and electrical property differences between deer antler EVs and impurities (DeCastro et al., 2021). However, variations in microfluidic chip design and operational parameters lead to significant variability in research outcomes. Differences in channel design, fluid velocity, and electric field strength result in varying shear forces and electrical stresses on EVs. Excessively high electric fields can induce EV membranes’ perforation, leading to the loss of endogenous bioactive molecules such as growth factors and enzymes, thereby reducing the tissue repair activity of deer antler EVs (Lu et al., 2019). Inconsistent chip material selection affects the degree of non-specific binding between EVs and channels, thereby altering EV recovery rates and purity (Guo et al., 2018). Currently, the lack of unified standards for microfluidic chip design and operational parameters in deer antler EVs isolation makes results difficult to replicate across laboratories. This limits the method’s application in comparative studies and constrains the realization of its precise separation advantages due to insufficient methodological standardization.

## 4 Deer antler EVs

**FIGURE 2:**
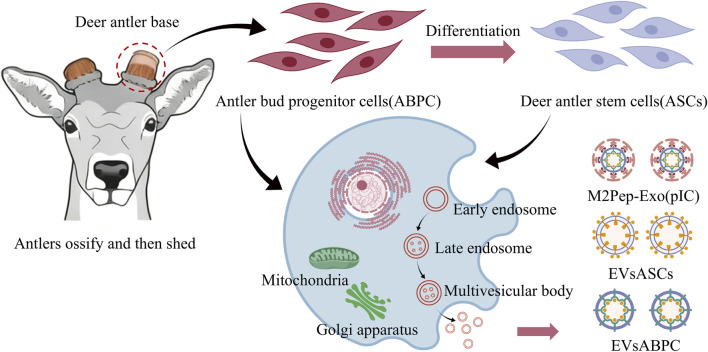
Schematic diagram of the release process of EVs from deer antler.

EVs serve as nanoscale messengers in intercellular communication. By transporting bioactive molecules such as proteins, nucleic acids, and lipids, they play a pivotal role in cellular signaling and material exchange. EVs have emerged as a research hotspot for diagnostic biomarkers, tissue repair tools, and drug delivery vehicles (Mondal et al., 2023). Their functionality is largely determined by the characteristics of their parent cells. Leveraging the unique biological advantages of deer antler, deer antler-derived EVs represent a highly active and distinctive branch within the EV field. To date, deer antler-derived EVs are primarily categorized into natural and engineered types. Natural types include antler bud progenitor cell-derived EVs (EVsABPC) and antler mesenchymal stem cell-derived EVs (EVsASCs). Engineered EVs are M2Pep/pIC-modified deer antler stem cell EVs (M2Pep-Exo (pIC)) (Figure 2). EVsABPC represent the most extensively studied and functionally diverse antler-derived EVs, originating from antler mesenchymal stem cells. These stem cells possess extraordinary regenerative capacity and are the only mammalian stem cells capable of driving complete, cyclical organ regeneration. Unlike conventionally sourced mesenchymal stem cells (bone marrow, adipose tissue, or umbilical cord-derived), which exhibit senescence after 10–15 culture passages, ABPCs maintain robust proliferation and regenerative capacity even after 50 passages (Hao et al., 2025).

The high activity of EVs makes them an ideal source for establishing stable parental cell lines (Yang et al., 2025b). EVsASCs originate from adipose-derived mesenchymal stem cells (ASCs) isolated from deer antler tissue, differing from the parental cells of EVsABPC. EVsABPC, validated by NTA and transmission electron microscopy, exhibit diameters comparable to EVsASCs, displaying typical cup-shaped or spherical structures. They express common EV markers, including Alix, TSG101, and CD81, with no exogenous modifications and preserved native vesicle integrity. EVsASCs, however, originate from deer antler adipose-derived mesenchymal stem cells (ASCs). The parental cells exhibit spindle-shaped vortex-like arrangements, highly express CD44 and CD90, and can differentiate into adipocytes, osteoblasts, and chondrocytes. They similarly possess the aforementioned core characteristics of EVs (Zuo et al., 2024). By conjugating M2 macrophage-targeting peptides and encapsulating poly (I: C), EVsASCs can be engineered into functional antitumor EVs M2Pep-Exo (pIC), synergistically enhancing antitumor efficacy (Zhang et al., 2021b).

EVsABPC significantly enriched proteins regulating organ regeneration, including cell cycle regulators (Cyclin D1, CDK2), tissue repair factors (IGF-1, TGF-β1), and stemness-maintaining proteins, while exhibiting higher expression of anti-inflammatory-related proteins compared to EVsASCs; EVsASCs, conversely, exhibit enrichment in immunoregulatory proteins (CD44, HSP70), lipid metabolism-related proteins, and cell adhesion molecules (Liu et al., 2022a). Their expression levels of differentiation-inducing proteins (RUNX2, PPAR-γ) are significantly higher than those in EVsABPC, reflecting the inheritance of parental cell differentiation potential. Common proteins between the two groups primarily comprised exosome structural proteins such as the membranin family, while differentially expressed proteins were predominantly enriched in pathways regulating regeneration, immune response, and metabolism (Yang et al., 2025a). Additionally, EVsABPC exhibited significantly higher expression abundance of regeneration-related miRNAs and long non-coding RNAs, capable of targeting cell proliferation and tissue regeneration pathways. EVsASCs, conversely, are enriched with immunoregulatory RNA molecules and mRNA transcripts involved in lipid metabolism and cellular differentiation. Specifically, the RNA profile of EVsABPC emphasizes maintaining stem cell characteristics, while the RNA molecules in EVsASCs play a dominant role in regulating immune cell activity and extracellular matrix remodeling (Fan et al., 2020).

## 5 Mechanism of action of deer antler EVs in promoting tissue repair

As a targeted signal carrier, velvet antler EVs activate the downstream core signaling pathway network by delivering bioactive molecules, thereby regulating the two major cell populations of stem cell function and immune microenvironment, and ultimately promoting the sequential process of tissue repair from inflammation regression to regeneration and reconstruction to functional homeostasis (Raghav et al., 2022). Moreover, bidirectional feedback exists between these two major cellular populations, with signaling pathways serving as the continuous molecular bridge throughout. Together, they form a closed-loop system encompassing the carrier, signals, cells, and tissues (Bjørge et al., 2017; Raghav and Mann, 2024).

### 5.1 Bioactive molecules derived from deer antler EVs

**FIGURE 3:**
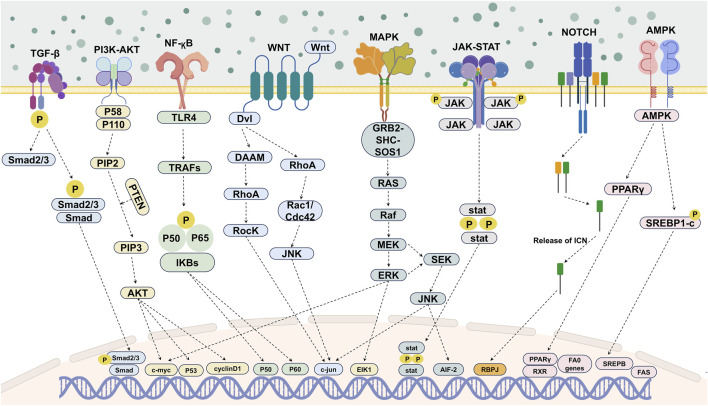
Schematic diagram of the multi-pathway regulatory model mediated by deer antler EVs promoting tissue repair (Note: In addition to describing the primary signalling pathways with direct effects, this article also presents other indirect or potentially interconnected signalling pathways in the diagram).

The tissue repair effects of deer antler EVs are mediated through the regulation of core intracellular signaling pathways by their enriched bioactive cargo (Figure 3). These EVs, acting as carriers for targeted delivery of bioactive molecules, construct a complex regulatory network that guides cellular behaviors essential for repair (Huynh, 2025; Raghav and Mann, 2021). Such cargoes constitute their core distinguishing feature from BMSC- or ADSC-derived EVs and form the material basis for their specific tissue repair functions (Table 2). Regarding specific growth factors, the most representative unique cargoes in antler EVs are Prkar2a and Galectin-1. Prkar2a is a high-abundance mRNA-encoded product unique to ABPCs-derived EVs. It significantly enhances the proliferation capacity of senescent stem cells and inhibits adipogenic differentiation by regulating cell cycle processes and core inflammatory pathways. Knocking down this molecule substantially weakens the anti-aging repair efficacy of EVs. Galectin-1, a core regulatory protein specifically secreted by antler stem cells, promotes vascularized cartilage formation by modulating PI3K-AKT and TGF-β pathways, thereby providing nutritional supply and structural support for tissue repair (Table 3). Regarding the miRNA profile, deep sequencing studies confirmed the presence of 399 miRNAs in Chinese red deer antler EVs, including 54 novel antler-specific miRNAs. Although these miRNAs exhibit low expression levels, they can target and regulate genes associated with rapid organ regeneration (Hao et al., 2025; Mahmud, 2021). Additionally, antler EVs enrich miR-1, miR-18a, and miR-18b. Their expression profiles significantly differ from those enriched in human BMSCs EVs, such as hsa-miR-128-3p and miR-26a. By targeting and suppressing the expression of aging-related genes, these miRNAs can reverse stem cell senescence phenotypes and maintain stem cell pluripotency (Jia et al., 2021; Lu et al., 2020). Notably, even miR-21-5p—shared with human BMSCs/ADSCs EVs—exhibits high abundance in deer antler EVs (Zhao et al., 2025a; Rawat et al., 2021) (Table 4). It demonstrates significantly superior efficacy in regulating inflammation resolution via the STAT3 pathway, with its target regulatory network encompassing multiple deer antler regeneration-specific sequences. This characteristic further reinforces its tissue repair specificity advantage (Raghav et al., 2023).

**TABLE 2:** Analysis of physicochemical characteristics and biological functions of EVs from different sources.

Comparison dimensions	Deer antler EVs	BMSC EVs	AMSC EVs	Key differentiation conclusions
Biological characteristics	Periodically proliferating cells originating from the perichondrium or mesenchymal zone of antler velvet exhibit dynamic expression patterns corresponding to the antler growth cycle. Primarily consisting of small EVs, these vesicles display high surface expression of antler-specific markers, with their quantity regulated by the antler growth stage.	Derived from quiescent MSCs in the bone marrow stroma, requiring *in vitro* induction for proliferation and secretion. Marked by universal MSC surface markers, exhibiting stable *in vitro* culture systems without significant periodic fluctuations.	Derived from adipose tissue matrix MSCs, exhibiting high *in vitro* expansion efficiency. Highly express adipogenic differentiation markers on the membrane surface. Abundant source, with significantly higher *in vitro* yield compared to BM-MSC-EVs.	Unique: Periodic secretion pattern + deer antler-specific membrane markers. Common: Both primarily secrete small/medium EVs and express universal EV markers.
Molecular cargo characteristics	1. Nucleic acids: Enriched with miRNA specific to deer antler, targeting regulation of the G1/S phase transition in the cell cycle; contains lncRNA associated with deer antler growth, regulating osteogenic/chondrogenic differentiation 2. Proteins: Highly express rapidly proliferating cell-associated factor and contain keratin family proteins involved in extracellular matrix remodeling. 3. Lipids: Rich in sphingomyelin, enhancing the efficiency of membrane fusion between EVs and target cells.	1. Nucleic acids: Enriches universal regeneration-related miRNAs, targeting apoptosis inhibition and angiogenesis promotion; lacks tissue-specific lncRNAs. 2. Proteins: Expresses classic MSC secreted proteins, primarily anti-inflammatory and homeostasis-restoring. 3. Lipids: Primarily phosphatidylcholine, with moderate membrane fusion efficiency.	1. Nucleic acids: Enriched with adipogenesis-related miRNAs regulating lipogenesis and angiogenesis; contains metabolism-related lncRNAs 2. Proteins: Highly express adipose-derived factors with dual anti-inflammatory and metabolic regulatory functions; VEGF content exceeds that of BM-MSC-EVs 3. Lipids: Rich in triglycerides with strong membrane stability but lower fusion efficiency compared to deer antler EVs.	Unique: Deer antler-specific nucleic acids (miRNA/lncRNA) + rapid proliferation-associated protein subtypes. Common: Both contain universal regenerative/anti-inflammatory factors such as VEGF and TGF-β.
Regeneration-related functions and mechanisms	1. Core function: Rapidly induces proliferative repair of damaged tissues, initiating *in situ* regeneration of bone/cartilage. 2. Mechanism of action: Continuously activates the Wnt/β-catenin pathway, promoting target cells into the S phase of the cell cycle - secretes keratin-associated proteins to construct a temporary extracellular matrix scaffold, guiding directed cell migration - low immunogenicity, with no significant immune rejection response.	1. Core function: Steady-state repair and anti-inflammation, suitable for repairing chronically damaged tissues. 2. Mechanism of action: Temporarily activates the Wnt/β-catenin pathway, primarily inhibiting target cell apoptosis, but with weaker proliferation-inducing capacity than deer antler velvet EVs.	1. Core function: Soft tissue repair and metabolic regulation, suitable for fat defects and skin wound repair. 2. Mechanism of action: Activates the PI3K/Akt pathway, promotes angiogenesis and adipocyte differentiation, exhibits stronger anti-inflammatory capacity than BM-MSC-EVs, but weaker osteogenic differentiation induction ability.	Unique: Persistent Wnt pathway activation + proliferative repair mode + matrix scaffold-guided function. Common: Activation of angiogenesis pathways, regulation of macrophage polarization, low immunogenicity.
Therapeutic application potential and limitations	Advantages: 1. Rapid regenerative repair for extensive bone defects and full-thickness cartilage injuries. 2. Compatible with scaffold materials to construct tissue engineering scaffolds with active proliferation induction capabilities. Limitations: 1. Availability constrained by deer antler growth cycles, with significant challenges in standardized isolation. 2. Insufficient depth in molecular mechanism research and limited clinical translation data	Advantages: 1. Steady-state repair of chronic degenerative diseases (e.g., osteoarthritis, diabetic wounds). 2. Stable source with mature *in vitro* expansion technology. Limitations: 1. Weak proliferation-inducing capacity, unsuitable for acute extensive injuries. 2. Significant functional decline in EVs derived from elderly donors.	Advantages: 1. Efficient repair of adipose tissue defects and skin wounds 2. Abundant sources, high *in vitro* yield, and easy to scale up for production Limitations: 1. Weak osteogenic/chondrogenic differentiation induction capacity, limiting application in hard tissue repair 2. Susceptible to donor metabolic status (e.g., dysfunctional EVs from obese donors)	Specific application scenario: Rapid regeneration of acute extensive hard tissue injuries common application scenario: Tissue repair associated with chronic inflammation
Clinical translation	Basic research phase: Efficacy validated only in animal models (rat bone defects, rabbit cartilage injuries), with no clinical trial data available.	Clinical phase I/II: Clinical trials for osteoarthritis, myocardial infarction, and other conditions have been initiated, with safety preliminarily validated.	Phase I clinical trial: Conducting clinical trials for skin wound and fat defect repair, with mature large-scale production technology.	Unique: Significant clinical translation gap requires breakthroughs in standardized preparation techniques common: Both require addressing issues such as donor heterogeneity and batch consistency

**TABLE 3:** Functional analysis of EVs growth factors from different sources.

Specific factors	Structural and sequence differences	Functional features	Human homologue comparisons
Prkar2a	Key molecules in deer antler EVs show differences in phosphorylation sites compared to humans	Regulate the cell cycle, inhibit the p16INK4a/p21CIP1 senescence pathway, and reverse the senescent phenotype of stem cells.	Human BMSCs/ADSCs EVs contain extremely low levels and show no significant anti-aging effects.
Galectin-1	BMP-2/4/7 deer family variants: Signal peptide sequence differences lead to multi-fold enhancement of receptor affinity	Targeted activation of the smad1/5/8 pathway, inhibition of Wnt pathway inhibitors such as DKK1, accelerates callus formation.	Human BMP-2: Requires high concentrations to be effective and may induce heterotopic ossification.
IGF-1	Only 68% homology with human IGF-1, with significant differences in the C-terminal domain.	Directly drive mesenchymal cells toward chondrocyte/osteoblast-specific differentiation to promote bone growth.	Human IGF-1: Promotes only cell proliferation and matrix synthesis, lacking the ability to induce rapid, directed differentiation.
HGF	Compared to human HGF, the α-chain kringle domain exhibits three amino acid substitutions.	Simultaneously promoting angiogenesis and chondrocyte proliferation, providing nutrients and cellular sources for rapid growth.	Human HGF: Primarily promotes angiogenesis, with minimal effect on cartilage differentiation.
EGF	The N-terminal glycosylation site of EGF in deer antler EVs differs from that in human EGF, exhibiting two amino acid variations that alter the conformation of the receptor-binding domain.	Synergistically enhances osteoblast proliferation and matrix mineralization with IGF-1, accelerating bone trauma healing while suppressing inflammatory factor release to mitigate inflammatory responses during the repair process.	Human EGF: Primarily promotes epithelial cell proliferation, exhibits weak directional regulatory effects on osteoblasts, and lacks anti-inflammatory synergistic effects.
VEGF	The heparin-binding domain of deer antler VEGF contains four amino acid substitutions, resulting in significantly enhanced dimer stability compared to human VEGF.	Efficiently induces vascular endothelial cell migration and luminal formation, providing ample blood supply for rapid antler growth while promoting paracrine interactions between osteoblasts and vascular endothelial cells to accelerate bone-vascular coupling regeneration.	Human VEGF: Exhibits lower angiogenesis efficiency, high concentrations readily induce vascular leakage, and has limited regulatory effects on bone-vascular coupling.
FGF	As a variant belonging to the FGF-2 family, the signal peptide cleavage site of FGF in deer antler EVs differs from that of human FGF-2. It exhibits 72% homology with human FGF-2 and demonstrates threefold enhanced receptor binding affinity.	Targeted activation of the FGFR1/2 pathway in fibroblasts and osteoprogenitor cells promotes collagen synthesis and bone callus remodeling, enhances the mechanical strength of bone tissue, and simultaneously delays osteoblast senescence.	Human FGF-2: Exhibits broad-spectrum activity with low specificity, readily inducing excessive fibrous tissue proliferation, and demonstrates limited anti-aging effects on osteoblasts.
TCTP	The C-terminal regulatory domain of TCTP in deer antler EVs exhibits three amino acid deletions, resulting in a more stable anti-apoptotic domain compared to human TCTP.	Inhibits osteocyte apoptosis, enhances stem cell pluripotency maintenance, improves cellular energy metabolism by regulating mitochondrial function, and assists in reversing aging-related metabolic disorders.	Human TCTP: Exhibits weak anti-apoptotic activity, has limited role in maintaining stem cell pluripotency, and lacks significant function in regulating mitochondrial metabolism.
S100A4	The calcium-binding site of deer antler EV S100A4 exhibits two amino acid substitutions, showing 75% homology with human S100A4 and enhanced binding specificity to the target protein.	Promote osteoblast migration and bone matrix deposition, regulate osteoclast activity balance, maintain bone remodeling homeostasis, and accelerate fracture healing processes.	Human S100A4: Primarily involved in tumor metastasis and tissue remodeling, with limited specificity in regulating bone metabolism, making it prone to disrupting normal bone homeostasis.
c-Myc	The transcription activation domain of c-Myc in deer antler EVs exhibits five amino acid mutations. Compared to human c-Myc, the degradation signal sequence is altered, resulting in an extended half-life.	Moderately activates stem cell proliferation-related genes (cyclin D1, CDK4) to promote mesenchymal stem cell expansion while maintaining their multipotent differentiation potential, with no carcinogenic risk.	Human c-Myc: Overactivation readily leads to cellular carcinogenesis and disrupts the balance between proliferation and differentiation potential, limiting its applications.
VWF	The A1 domain of VWF in deer antler EVs harbors three amino acid substitutions. Compared to human VWF, the platelet-binding site exhibits optimized conformation and enhanced adhesion activity.	Enhances adhesion between vascular endothelial cells and platelets, promotes hemostasis and coagulation while facilitating vascular repair, provides a stable microenvironment for bone trauma sites, and aids in the early formation of callus.	Human von Willebrand factor (VWF): Primarily involved in the hemostasis process, with minimal synergistic effects on vascular repair and bone callus formation, and is readily degraded by proteases.
TIMP	Belonging to the TIMP-2 subtype, the metalloproteinase-binding domain of TIMP in deer antler EVs exhibits two amino acid mutations, resulting in a broader inhibitory spectrum compared to human TIMP-2.	Specifically inhibits the activity of matrix metalloproteinases such as MMP-2 and MMP-9, reduces bone matrix degradation, protects newly formed bone tissue, promotes osteoblast differentiation, and suppresses excessive osteoclast activation.	Human TIMP-2: Exhibits low inhibitory specificity and weak regulatory effects on osteoclasts, failing to effectively balance bone formation and resorption.
COL2A1	The triple-helix core domain of COL2A1 in deer antler EVs exhibits three amino acid substitutions, sharing 78% homology with human COL2A1. Hydroxylation modification sites are optimized, enhancing intrafibrillar cross-linking efficiency by twofold.	Specifically promotes chondrocyte synthesis of type II collagen and proteoglycans, maintaining cartilage tissue elasticity and load-bearing capacity. Synergizes with Galectin-1 to accelerate the conversion of cartilage to bone tissue within the callus, providing a stable cartilage transition layer for bone repair.	Human COL2A1: Exhibits low fibrillar cross-linking efficiency, with its chondrocyte-promoting effects dependent on exogenous factor co-regulation. During bone trauma repair, it demonstrates slow cartilage regeneration rates and is susceptible to degradation by MMPs, failing to maintain long-term stability of the cartilage transition zone.
COL6A1	The triple-helix domain of COL6A1 in deer antler EVs exhibits four amino acid substitutions. Compared to human COL6A1, it demonstrates enhanced fibrillar assembly efficiency and increased elasticity.	As a component of the bone matrix scaffold, it promotes osteoblast adhesion and spreading, enhances the toughness and stability of the bone matrix, and synergizes with other factors to accelerate bone tissue regeneration and repair.	Human COL6A1: Slow fibrillar assembly, poor elasticity, limited promotion of osteoblast adhesion, weak bone repair support effect.

**TABLE 4:** Functional analysis of EVs-derived miRNAs from different sources.

Deer antler EVs specific miRNA	Core target genes	Functional effects	Human BMSCs/ADSCs EV correspondence
miR-143-3p	Prrx1, DKK1, SOX9	Maintain mesenchymal stem cell homeostasis, promote chondrogenic differentiation, and inhibit Wnt pathway negative regulators.	Low expression, with target genes biased toward adipogenic differentiation regulation
miR-221-5p	p27Kip1, PTEN	Promote the G1/S transition in the cell cycle to accelerate the proliferation of mesenchymal cells.	Expression functions primarily to inhibit tumor cell proliferation, not regeneration.
miR-34a-5p	Notch1, SIRT1	Regulate the balance between stemness and differentiation in stem cells to promote rapid osteogenesis.	In human EVs, it primarily functions as a tumor suppressor, inhibiting cell proliferation.
miR-140-3p	Prrx1, MMP13	Forms a negative feedback loop with Prrx1 to regulate rapid chondrogenesis and inhibit cartilage degradation.	Human EVs show low to moderate expression and have limited chondroprotective effects.
miR-PC-2869	138 potential targets enriched in the bone mineralization pathway	Promote proliferation of deer antler mesenchymal cells and inhibit excessive differentiation of prechondrocytes	This miRNA is completely absent in humans.
miR-21-5p	PTEN, Sprouty1, PDCD4	Promote the osteogenic differentiation of mesenchymal stem cells, inhibit cell apoptosis, and enhance tissue repair capacity.	Moderate expression, with target genes predominantly involved in damage repair regulation and weaker regeneration-related functions.
miR-125b-5p	BMPR2, EGFR, Lin28	Maintain stem cell pluripotency, promote cartilage matrix synthesis, and inhibit chondrocyte hypertrophy.	Low expression, functionally biased toward inflammation regulation, with no significant effects on cartilage regeneration.
miR-155-5p	SOCS1, CEBPB, SHIP1	Modulate the immune microenvironment, inhibit adipogenic differentiation, and assist osteogenic signaling pathways.	High expression, core function biased toward immune regulation, not dominated by regeneration
miR-199a-3p	Notch3, VEGF, FOXO1	Promote angiogenesis, enhance the directed differentiation of mesenchymal cells into osteoblasts, and synergistically facilitate bone regeneration.	Moderate expression, weakly associated with bone repair, with target genes biased toward vascular homeostasis regulation.
miR-200c-3p	ZEB1, E-cadherin, BMI1	Inhibit epithelial-mesenchymal transition, maintain stable mesenchymal cell phenotype, and mildly promote cartilage repair.	Highly expressed, functionally focused on maintaining tissue homeostasis, with unclear active regeneration orientation.
circRNA2829	MEK1, ERK2, Smad3	Regulates the MAPK/Smad signaling pathway to promote proliferation of antler mesenchymal cells and deposition of cartilage matrix, enhancing proliferative activity and differentiation stability in regenerated tissues.	Human EVs exhibit extremely low expression levels, lack clear regenerative functions, and target genes are biased toward regulating cellular stress responses.
lncRNA-ABPC1	Runx2, COL2A1, miR-320a	Adsorption-inhibited regenerative miRNA releases functional suppression, promotes balanced osteogenic-chondrogenic differentiation, inhibits premature chondrocyte hypertrophy, and maintains the integrity of regenerative cartilage tissue.	Human EVs show low to moderate expression, with core functions leaning toward cartilage matrix metabolism regulation and no significant regenerative orientation.

### 5.2 Molecular and signaling pathway regulatory mechanisms

#### 5.2.1 Wnt/β-catenin pathway

**FIGURE 4:**
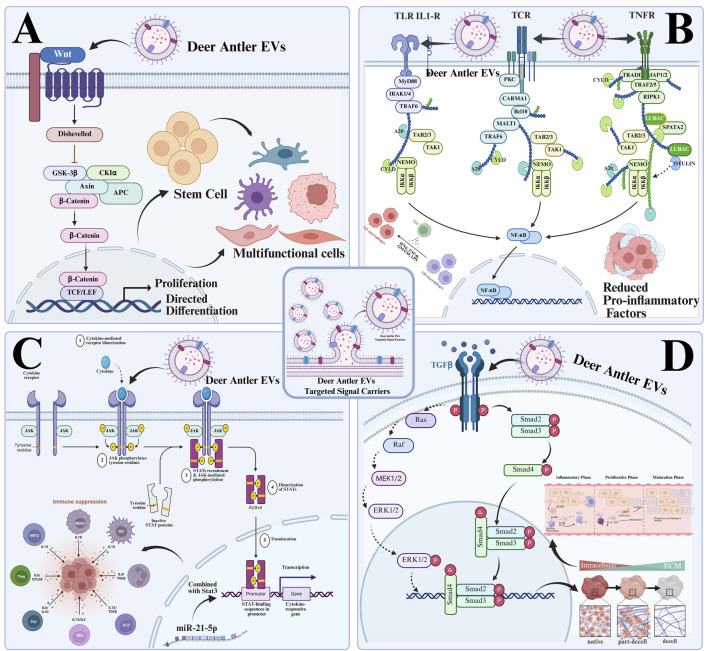
Simulation of biological processes mediated by deer antler extracellular vesicles promoting tissue repair through core pathways. **(A)** Modulating the Wnt/β-catenin signaling pathway to promote stem cell proliferation and differentiation. **(B)** Modulating the NF-κB signaling pathway to regulate immune inflammation. **(C)** Modulating the miR-21-5p/STAT3 signaling pathway to regulate immune suppression. **(D)** Modulating the TGF-β signaling pathway to promote functional homeostasis restoration during tissue regeneration.

The Wnt/β-catenin pathway is a key signalling pathway regulating cell proliferation, differentiation, apoptosis, and tissue homeostasis (Ma et al., 2023; Zhang et al., 2023). In the inhibited state (absence of Wnt ligands), β-catenin is phosphorylated by the degradation complex and degraded by the proteasome, silencing target genes (such as c-Myc, Cyclin D1, Runx2), maintaining cellular quiescence, or differentiation homeostasis (Choi et al., 2017; Raghav and Mann, 2021). In the activated state (Wnt ligand binding), the Wnt ligand binds to Frizzled/LRP5/6, inhibiting the activity of the degradation complex. β-Catenin translocates to the nucleus, forms a complex with TCF/LEF, activates target gene expression, and regulates cell proliferation and differentiation (Figure 4A) (Mahoney et al., 2022).

##### 5.2.1.1 Regulatory mechanisms of the Wnt/β-catenin pathway in tissue repair

Activation of the Wnt/β-catenin pathway initiates tissue repair processes by regulating immune cell function and inflammatory cytokine secretion (Robinson et al., 2020; Blyszczuk et al., 2017). Wnt3a activates β-catenin, upregulating IL-10 and TGF-β expression in macrophages while suppressing TNF-α and IL-1β secretion, thereby antagonizing the canonical NF-κB pathway and accelerating inflammation resolution (Xie et al., 2020). Nuclear translocation of β-catenin promotes NET formation, facilitating efficient clearance of necrotic tissue and pathogens (Jiang et al., 2021). Activation of the Wnt/β-catenin pathway also directly upregulates Runx2 expression via TCF/LEF, while STAT3 is activated through miR-21-5p-mediated SOCS3 inhibition. STAT3 binds Runx2 to enhance its transcriptional activity, exerting synergistic effects to promote stem cell osteogenic differentiation. Regulation of stem cell proliferation is linked to the NF-κB pathway, where both NF-κB and Wnt/β-catenin pathways upregulate c-Myc and Cyclin D1. This ensures a stable supply of MSCs and ESCs, maintaining osteogenic differentiation potential while enhancing cell proliferation. It also stimulates ESC proliferation to accelerate wound re-epithelialization (Feng and Meng, 2021). Furthermore, ECM remodeling is a central component of tissue repair, jointly regulated by two systems: synthesis and proteolytic degradation. During this process, TGF-β promotes collagen and fibronectin synthesis, while Wnt/β-catenin downregulates MMP-2 and MMP-9 to prevent excessive ECM degradation. Concurrently, it stimulates Col1A1 and fibronectin synthesis to construct a stable extracellular matrix scaffold (Liu et al., 2023).

Vesicles in deer antler mesenchymal stem cell conditioned medium serve as potent activators of the Wnt pathway, significantly activating the Wnt/β-Catenin pathway. This leads to an 8-fold increase in Wnt-3a mRNA levels and a 4-fold increase in Wnt-10b mRNA levels. LEF1 (a classic downstream factor of the Wnt pathway) increased 5.6-fold, promoting hair growth and skin wound healing (Seo et al., 2018). Bone marrow-derived MSCs, when stimulated by ALB enzymatic digestion, produce Exos with enhanced capabilities to promote bone and vascular regeneration. ALB enzymatic digestion enhances regenerative function by upregulating miR-21-5p expression within Exos. These differentially expressed genes are closely associated with multiple biological processes, including vesicle-mediated transport and Wnt signalling pathways (Zuo et al., 2024).

#### 5.2.2 NF-κB pathway

The NF-κB pathway is primarily activated through two main routes: the canonical pathway (dependent on IKKβ/NEMO) and the non-canonical pathway (dependent on IKKα) (Hossain et al., 2018; Polley et al., 2016). The canonical pathway relies on inflammatory signals to induce IKKβ phosphorylation of IκBα, leading to IκBα ubiquitination and degradation (Liu et al., 2018). This allows p65/p50 to translocate to the nucleus, where they regulate the expression of pro-inflammatory factors and chemokines, initiating the inflammatory response. This pathway focuses on early-stage inflammation initiation and immune cell recruitment following tissue injury (Figure 4B) (Pradère et al., 2016). Receptor activation in the non-canonical pathway induces IKKα to phosphorylate p100, processing it into p52. This forms the p52/RelB dimer, which translocates to the nucleus to regulate lymphocyte development, B cell activation, and tissue remodelling. This pathway facilitates late-stage repair processes, such as angiogenesis and ECM remodeling (Verma, 2004).

##### 5.2.2.1 Regulatory mechanisms of the NF-κB pathway in tissue repair processes

The NF-κB pathway plays a double-edged role in tissue repair. Moderate activation serves as a promoter of the injury response, initiating repair by triggering inflammation, clearing damaged tissue, and regulating cell proliferation (Liu et al., 2022b). However, excessive or sustained activation becomes an inhibitor of repair, leading to chronic inflammation persistence, promoting fibrosis, and suppressing stem cell function, ultimately resulting in repair failure (Li et al., 2025b). The NF-κB pathway is antagonized by the miR-21-5p/STAT3 axis, where STAT3 blocks excessive nuclear translocation of p65 by inhibiting IκBα degradation, thereby balancing pro-inflammatory and anti-inflammatory responses. During the initial phase of tissue injury, rapid activation promotes expression of target genes, including proinflammatory factors, chemotactic molecules, and adhesion molecules, thereby recruiting neutrophils and macrophages to the injury site (Kanaji et al., 2011). Excessive activation downregulates key osteogenic and chondrogenic transcription factors, inhibiting MSC osteogenic/chondrogenic differentiation while promoting adipogenic differentiation, thereby delaying bone defect repair (Liu et al., 2021). Notably, the non-canonical NF-κB pathway (p52/RelB) participates in extracellular matrix remodeling and tissue functional maturation; its insufficient activation leads to disorganized repair tissue architecture (Takakura et al., 2020). During remodeling, it synergizes with the TGF-β1/Smad pathway to form the NFκB–TGF-β1–ATF-4 axis. NF-κB serves as the key integrator in this axis, regulating TGF-β1 expression (Deng et al., 2021). TGF-β1 activates ATF-4 via Smad3 and p38/ERK pathways, thereby promoting fibroblast proliferation, adhesion, and migration, facilitating macrophage polarization toward the M2 type, and accelerating tissue healing. Additionally, NF-κB synergistically drives epithelial cell migration and proliferation with the EGFR pathway, promoting repair and regeneration of mucosal tissues such as the intestine and cornea (Zou et al., 2023).

Toll-like receptors play a crucial role in inflammatory and immune responses in CPB rats. Compared to the CPB group, the EXO group exhibited significantly reduced levels of TLR2, TLR4, MyD88, and NF-κB, indicating that adipose-derived mesenchymal stem cell-derived EVs can reverse the expression of TLR2/TLR4 signaling pathway–related proteins in CPB rats. Concurrently, following activation of the TLR2/TLR4 signaling pathway, EVs failed to reduce levels of IL-1β, IL-6, tumor necrosis factor-α, and malondialdehyde, nor did they elevate levels of IL-10, superoxide dismutase, and nitric oxide. Thus, AMSC-derived EVs alleviate postoperative cognitive impairment in CPB rats via the TLR2/TLR4 signaling pathway (Yang et al., 2020a). Restoring periodontal homeostasis and promoting periodontal bone repair/regeneration represent major challenges in treating diabetic periodontitis. ASC-exos exhibit dual regenerative effects in this process: they restore the osteogenic potential of resident mesenchymal stem cells by reversing high glucose (HG)-induced inhibition of proliferation and migration, more importantly, enhancing cell survival, reducing cell death, and strengthening differentiation toward the osteoblastic lineage under HG conditions. They also effectively clear HG-induced excessive ROS production to alleviate inflammation, and efficiently inhibit HG treatment-induced p65 translocation and upregulation, blocking the NF-κB transduction pathway, thereby reducing the M1/M2 macrophage ratio (Guo et al., 2025a).

#### 5.2.3 miR-21-5p/STAT3 pathway

miR-21-5p is a highly conserved member of the microRNA (miRNA) family that mediates post-transcriptional regulation by binding to the 3′untranslated region (3′UTR) of target gene mRNAs. It primarily exerts anti-inflammatory, pro-proliferative, anti-apoptotic, and pro-angiogenic effects in tissue repair (De Santi et al., 2017). STAT3 is the core effector molecule of the JAK/STAT pathway. The primary interaction mode between the miR-21-5p/STAT3 axis is a positive feedback loop, mediated through two distinct pathways (Qin et al., 2023). Indirect activation: miR-21-5p targets and suppresses negative regulators of STAT3 (SOCS3, PTEN, PDCD4), thereby releasing inhibition on STAT3. This promotes its phosphorylation and nuclear translocation, amplifying the pro-repair signals of the STAT3 pathway (Qin et al., 2025). Direct regulation: miR-21-5p directly binds the 3′UTR of STAT3 mRNA to suppress its expression (Figure 4C). However, in tissue repair, activation predominates to prevent fibrosis or tumour risks associated with STAT3 overactivation (Li et al., 2021).

##### 5.2.3.1 Regulatory mechanisms of the miR-21-5p/STAT3 pathway axis in tissue repair

During tissue repair, miR-21-5p regulates the resolution of inflammation and immune homeostasis by inhibiting SOCS3, activating STAT3, promoting M2 macrophage polarization, upregulating anti-inflammatory factors such as IL-10 and TGF-β, and simultaneously suppressing pro-inflammatory factors like TNF-α and IL-1β, thereby mitigating excessive inflammation (Ji et al., 2020; Bi et al., 2020). In a cerebral ischemia-reperfusion injury model, miR-21-5p overexpression inhibits STAT3 activation, thereby promoting PI3K/AKT signaling, increasing the Bcl-2/Bax ratio, reducing neuronal apoptosis, and decreasing cerebral infarction volume. The miR-21-5p/STAT3-ERK axis upregulates GAP-43 expression, promoting axonal regeneration and myelination, and accelerating repair of sciatic nerve defects in rats (Hou et al., 2018). In skin wound healing, miR-21-5p targets STAT3 to activate the PI3K/AKT pathway, increasing VEGF secretion by endothelial cells and enhancing neovascular density, thereby accelerating granulation tissue formation. Targeting STAT3 activates ERK1/2, boosting keratinocyte proliferation and migration to hasten wound re-epithelialization (Yang et al., 2024). Furthermore, miR-21-5p inhibits activation of renal tubulointerstitial fibroblasts via the STAT3-Smad7 axis, reducing renal tissue fibrosis. The miR-21-5p/STAT3-PI3K/AKT axis also upregulates RUNX2 expression, promoting osteogenic differentiation of BMSCs and accelerating bone defect repair (Ye et al., 2017).

AMSC-Exos demonstrate significant potential in skin wound healing. It effectively promotes proliferation and migration of HACAT corneal carcinoma cells, while enhancing migration and tubulogenesis in HUVEC endothelial cells. In a mouse model of skin injury, AMSC-Exo treatment improves wound healing quality by stimulating angiogenesis, regulating extracellular matrix deposition, and promoting re-epithelialization. Notably, miR-21-5p within AMSC-Exo downregulates MMP1 production and reduces scar formation by modulating STAT3 and TIMP3 expression (Meng et al., 2024).

#### 5.2.4 TGF-β signaling pathway

The TGF-β (Transforming Growth Factor–β) superfamily constitutes a pivotal signalling network regulating tissue homeostasis, repair, and regeneration. Its core members include three subtypes—TGF-β1, TGF-β2, and TGF-β3—along with branches such as BMPs and Activin (Hanna and Frangogiannis, 2019). TGF-β1 initiates early in injury, peaks during mid-repair, and persists in late stages. It promotes inflammation resolution, ECM synthesis, and fibroblast proliferation (Yang et al., 2021a). Moderate activation facilitates repair, while excessive activation leads to scarring/fibrosis (Willenborg et al., 2025). TGF-β2 exhibits low expression throughout repair, primarily enriched in epithelial tissues. It regulates epithelial cell differentiation, inhibits excessive angiogenesis, and maintains epithelial barrier function (Figure 4D). Overexpression may delay wound healing (Zhao et al., 2025b). TGF-β3 initiates mid-repair and exhibits high expression in the late phase. It promotes ECM remodelling, inhibits myofibroblast transformation, counters scarring by antagonizing TGF-β1, and maintains repair equilibrium, making it a key subtype for scar-free regeneration (Lee et al., 2023). Activated TGF-β dimers bind to TβRII, recruiting and phosphorylating TβRI. Phosphorylated TβRI corresponds to Smad (Smad2/3 or Smad1/5/8), forming Smad–Smad4 complexes that translocate to the nucleus to regulate target gene expression (DiNicolantonio et al., 2021).

##### 5.2.4.1 Regulatory mechanisms of the TGF-β pathway in tissue repair processes

TGF-β1 activates VEGF and angiopoietin-1 expression via Smad3, synergistically promoting endothelial cell migration and luminal formation with the non-canonical NF-κB pathway (Sun et al., 2018). During gastrointestinal mucosal injury, activation of the TGF-β/PI3K/AKT axis promotes intestinal epithelial cell proliferation and migration, accelerating mucosal wound healing. It also activates the Ras-Raf-MEK-ERK signaling axis, promoting fibroblast migration and collagen synthesis (Bao et al., 2022). Activation of the TGF-β/JNK pathway induces hepatocyte EMT, promotes hepatic stellate cell activation, and leads to excessive collagen deposition, triggering liver fibrosis (Zhang et al., 2018). Inhibiting the JNK pathway reverses this pathological process. Notably, phosphorylated Smad3 binds to β-catenin, forming a Smad3-β-catenin-TCF/LEF ternary complex that jointly binds target gene promoters to enhance transcriptional activity. It also reduces β-catenin ubiquitination and degradation by inhibiting GSK-3β activity, promoting its nuclear accumulation and intensifying Wnt pathway activation. This primarily manifests as synergistic activation of the TGF-β/Wnt pathways, upregulating osteogenesis-specific transcription factors to promote differentiation of BMSCs into osteoblasts, thereby accelerating callus formation and bone defect healing (Zhao et al., 2018). In fetal skin wounds, the synergistic action of TGF-β3 and the Wnt/β-catenin pathway results in a reticular arrangement of collagen fibers without excessive myofibroblast activation, achieving scar-free healing (Wu et al., 2025a).

AnSC-Exos significantly enhances the healing speed and quality of burn wounds. Following AnSC-Exos treatment, the relative mRNA expression levels of ColA2/Col3A1 and TGF-β1 were significantly elevated, while MMP3 levels were markedly reduced. This treatment also promotes proliferation and migration of human umbilical vein endothelial cells, modulates M2 macrophage polarization, stimulates angiogenesis, inhibits myofibroblast generation, and enhances collagen deposition, thereby accelerating burn wound repair (Guo et al., 2025a).

### 5.3 Stem cell regulation

MSCs are a type of adult stem cell originating from the mesoderm, possessing multipotent differentiation potential, immunomodulatory activity, and paracrine functions. They are widely distributed across various tissues (Montazersaheb et al., 2022). Examples include bone marrow-derived mesenchymal stem cells (BMSCs), adipose-derived mesenchymal stem cells (ADSCs), and umbilical cord-derived mesenchymal stem cells (Liu et al., 2022c). MSCs hold core application value in regenerative medicine, tissue engineering, and disease treatment, due to their multiple differentiation potentials, immunomodulatory activity, and ability to promote tissue repair by secreting bioactive factors. The development and repair of the nervous system depend on the critical role of neural mesenchymal stem cells. Following treatment of neural stem cells with either EVsABPC or EVsBMSC, the number of neural stem cells in the EVsABPC group increased by 1.3-fold and 2.1-fold compared to the EVsBMSC group and the blank control group (rapid medium), respectively. This indicates that EVsABPC possesses a stronger capacity to promote neural stem cell proliferation and neuroregeneration (Wu et al., 2023). In wound healing experiments, EVsABPCs transmitted ABPC bioactive signals to BMSCs, significantly enhancing BMSC proliferation, converting BMSCs into ABPC-like phenotype cells, and inducing BMSC differentiation toward a tissue-regenerative phenotype. Compared to PBS, EVsABPCs increased wound area recovery by 4.2-fold, significantly higher than the 1.8-fold increase observed with EVsBMSCs (Li et al., 2025a). Regarding anti-aging effects, EVsABPCs increased the number of 5-ethynyl-2′-deoxyuridine (EdU)-positive cells and the proportion of S-phase cells, significantly lengthened telomeres in aged bone marrow mesenchymal stem cells, and reduced their aging markers (Hao et al., 2025). Furthermore, ASC-Exos enhanced chondrocyte proliferation capacity as well as the proliferation and migration abilities of BMSCs. They upregulated mRNA and protein expression levels of aggrecan, type II collagen (COLII), and Sox9 in chondrocytes, significantly promoting the repair of cartilage tissue damage (Zhou et al., 2024).

### 5.4 Immune microenvironment remodeling

#### 5.4.1 Immune cell function and phenotypic conversion

The immune microenvironment is a dynamic and complex system formed locally within tissues by immune cells, chemokines, the extracellular matrix (ECM), metabolic products, and the vascular-nervous network (Petković et al., 2025; Wang et al., 2025b). It regulates immune cell proliferation, activation, and cytokine secretion, balancing innate and adaptive immune responses. Its core functions include maintaining tissue homeostasis, modulating inflammatory responses, and participating in tissue repair (Xiong et al., 2022). Following tissue injury, the immune microenvironment undergoes dynamic remodeling through processes such as inflammatory initiation, anti-inflammatory transition, and homeostasis restoration. During tissue regeneration, paracrine signals from stem cells and parenchymal cells reciprocally modulate the phenotype and function of immune cells, forming a bidirectional regulatory loop between immunity and regeneration (Cinat et al., 2021). Macrophages, as core cells of the immune microenvironment, exert significant influence on regenerative outcomes through phenotypic transitions. The traditional M1/M2 binary polarization framework provides a foundational paradigm for understanding their functions. Early M1-type macrophages eliminate pathogens and necrotic tissue by secreting TNF-α, IL-1β, iNOS, etc., while simultaneously promoting MSC proliferation and migration via the TNF-α/IL-6 pathway. Late-phase M2 macrophages suppress excessive inflammation by producing anti-inflammatory factors like IL-10, TGF-β, and Arg-1, while secreting growth factors such as VEGF and BMP-2 to regulate stem cell differentiation and tissue repair (Mantovani et al., 2013). However, this binary framework has faced widespread criticism in recent years (Jayasingam et al., 2019; Summer et al., 2025). Its limitations primarily lie in oversimplifying the phenotypic complexity of macrophages *in vivo*. The M1/M2 phenotypes defined by *in vitro* experiments are difficult to strictly distinguish *in vivo*, with numerous intermediate phenotypes exhibiting characteristics of both types actually existing. Moreover, phenotypic conversion is not a unidirectional irreversible process but a reversible one dynamically regulated by microenvironmental signals (Murray et al., 2014; Stout and Suttles, 2004). Simultaneously, the significance of *in vivo* immune heterogeneity remains underappreciated, extending across tissue, cellular, and molecular levels. At the tissue-specific level, macrophages in bone *versus* skin tissues exhibit marked differences in baseline phenotypes, functional preferences, and injury response patterns—for instance, bone macrophages are more responsive to osteogenesis-related signaling (Blériot et al., 2020; Pang et al., 2022). At the cellular level, macrophage subpopulations within the same tissue exhibit functional heterogeneity based on origin (embryonic or monocyte-derived) and differentiation stage. At the molecular level, concentration gradients of signals like ROS, lactate, and extracellular matrix fragments in the injured microenvironment differentially regulate macrophage polarization through pathways such as NF-κB and STAT6 (Sica and Mantovani, 2012). Imbalanced polarization prolongs inflammation and inhibits regeneration. However, the mechanisms underlying this imbalance exhibit significant individual variation and tissue specificity due to immune heterogeneity, making it impossible to fully explain solely through a binary framework.

Other immune cells also participate in tissue regeneration through synergistic interactions, and their functions are similarly regulated by immune heterogeneity. Early neutrophil infiltration clears debris and releases NETs, recruiting MSCs and macrophages via IL-8 and CXCL1 (Wan et al., 2020). However, NETs exhibit tissue-specific effects: they promote healing in skin wound repair but inhibit osteogenesis when excessively present in bone defects. Th2 cells secrete IL-4 and IL-13 to promote M2 polarization (Espinosa Gonzalez et al., 2022), while Tregs suppress excessive inflammation via IL-10 and TGF-β and enhance MSC osteogenic/chondrogenic differentiation. γδ T cells directly secrete BMP-2 to promote bone regeneration (Jiang et al., 2023), while NK cells enhance M1 activity via IFN-γ in the early injury phase and suppress inflammation to promote angiogenesis in the later phase (Zhang et al., 2019). The functions of these cells are regulated by local immune microenvironment heterogeneity and individual immune status.

#### 5.4.2 Cascade regulation of cytokine networks

The immune microenvironment regulates the regenerative process through a cascade conversion from proinflammatory factors to anti-inflammatory factors and then to growth factors (Guo et al., 2025c; Saldaña et al., 2019). However, following tissue injury, TNF-α and IL-1β activate local cells via the MAPK/NF-κB pathway, releasing chemokines to recruit immune cells and stem cells. TGF-β and IL-10 suppress proinflammatory factor expression by inhibiting the NF-κB pathway while activating the Smad2/3 pathway to promote M2 polarization and MSC osteogenic differentiation (Zuo et al., 2025; Bueno-Silva et al., 2020). VEGF, PDGF, BMPs, IGF-1, and others form a growth factor network that regulates angiogenesis, ECM synthesis, and stem cell differentiation, respectively (Wu et al., 2022). Furthermore, IL-6 exhibits dual functions: it promotes inflammation and stem cell proliferation via the STAT3 pathway during early stages, while later converting to anti-inflammatory signaling through sIL-6R to facilitate tissue repair (Yoo and Lee, 2023).

#### 5.4.3 The supporting role of microenvironmental homeostasis

During the inflammatory phase, immune cells primarily rely on glycolysis, with the resulting lactic acid promoting VEGF expression via the HIF-1α pathway, thereby enhancing angiogenesis (Wei et al., 2024). In the tissue repair phase, the metabolic profile shifts toward oxidative phosphorylation, characterized by elevated lactic acid levels and enhanced glutamine metabolism, providing energy and biosynthetic precursors for MSC differentiation (Neto et al., 2023). Additionally, MMPs secreted by immune cells degrade damaged ECM, releasing extracellular matrix fragments that serve as injury signals modulating immune cell polarization. Concurrently, M2 macrophages secrete components like fibronectin and collagen to construct new extracellular matrix scaffolds, providing physical support for stem cell adhesion, proliferation, and differentiation (Li et al., 2020; Selvaraj et al., 2024).

Multiple molecules associated with inflammatory response regulation were identified in EVsABPC, including VWF, TIMP, and COL6A1. Following spinal cord injury, EVsABPC treatment significantly attenuated the upregulation of proinflammatory cytokines in the lesion area, indicating its potent capacity to mitigate inflammatory responses and modulate M1/M2 macrophage polarization, thereby promoting cell survival and spinal cord repair (Yang et al., 2025b). Regarding inflammatory regulation during osteogenesis, EVsABPC treatment significantly reduced iNOS expression in macrophages while markedly increasing Arg1 expression. Furthermore, EVsABPC elevated IL-10 levels in BMSCs while decreasing TNF-α, PGE2, and IL-1β levels. This induced macrophage polarization from M1 to M2 type and mitigated inflammatory responses, potentially contributing to its significant beneficial effects on bone regeneration (Li et al., 2025a). Serum inflammatory markers associated with the senescence-associated secretory phenotype (SASP) serve as critical indicators of senescent states. In anti-aging studies involving aged mice and rhesus monkeys, EVsABPC, EVs-F-BMSC, and EVs-A-BMSC all reduced serum levels of IL-8, IL-6, IL-1β, and TNF-α in aged mice. Among these, EVsABPC exhibited the most pronounced anti-inflammatory effect, followed by EVs-F-BMSC, and then EVs-A-BMSC. This indicates that EVsABPC possesses a stronger capacity to reduce serum levels of aging-associated molecular markers and slow the rate of biological aging (Hao et al., 2025).

## 6 New trends in deer antler EVs promoting tissue repair

### 6.1 Engineering modification

Deer antler EVs possess inherent biocompatibility and low immunogenicity, establishing a solid foundation for therapeutic applications. However, their clinical translation faces two major bottlenecks: poor targeting and difficulty in controlling endogenous components. These issues can be addressed through experimentally validated engineering modification techniques. Current engineering strategies focus on three validated technical pathways. Regarding component engineering, experiments confirm that the molecular composition of deer antler EVs can be regulated through hypoxia pretreatment and gene editing techniques. For instance, *in vitro* studies demonstrate that hypoxia stimulation enhances the enrichment of the pro-angiogenic factor miR-210 within EVs, while targeted editing of the miR-29 gene significantly reduces the fibrotic tendency of recipient cells (Du et al., 2025). At the membrane modification level, chemical conjugation and membrane fusion techniques have successfully anchored RGD peptides to EVs. *In vivo* animal studies demonstrate that this modification significantly enhances EVs’ retention in cartilage defect sites while reducing leakage to non-target organs like the liver and spleen (Yang et al., 2021b). In the field of drug delivery engineering, electroporation has been validated as an efficient method for loading exogenous small-molecule drugs into EVs. This composite system demonstrated synergistic therapeutic effects in preliminary cellular experiments, achieving controllable drug loading efficiency while maintaining EVs’ membrane integrity (Kim et al., 2025a).

### 6.2 Novel delivery system

The transient retention time of EVs following a single injection is widely regarded as a technical bottleneck. The primary approach to addressing this issue should be the development of sustained-release systems based on established carrier technologies. Thermosensitive hydrogels have been demonstrated as highly effective EV reservoirs. In animal models of bone defect repair, thermosensitive hydrogels loaded with EVs gel *in situ* at body temperature and degrade in response to the defect site’s mildly acidic environment, enabling sustained EVs release. This system maintains effective therapeutic concentrations for over 7 days, significantly outperforming single-injection therapies (Xu et al., 2023). Another approach involves integrating EVs with 3D-printed bio-scaffolds (e.g., hydroxyapatite-collagen scaffolds). Existing studies confirm that embedding EVs into 3D-printed scaffolds not only provides physical support for bone defects but also enables sustained EV release during scaffold degradation. In rabbit femoral defect models, this composite system more effectively promotes osteoblast infiltration and angiogenesis compared to scaffolds alone (Vakilian et al., 2024).

### 6.3 Deepening of the mechanism of action

At the level of epigenetic regulation, existing studies have identified specific non-coding RNAs in deer antler EVs that can regulate DNA methylation and histone acetylation in recipient cells. For example, miR-140 in deer antler EVs has been demonstrated to target DNMT3A in chondrocytes, promoting cartilage differentiation by reducing methylation levels at the Sox9 gene promoter (Song et al., 2025). Subsequent studies may focus on validating these pathways in in vivo models to elucidate quantitative relationships between EV-derived non-coding RNAs and epigenetic modifications. Based on existing molecular clues in this direction, conventional epigenetic detection tools can be employed. Regarding immune microenvironment regulation, *in vitro* experiments confirm that deer antler EVs induce M1-M2 polarization in macrophages (Yang et al., 2025b). Preliminary data indicate that EV-derived TNF-α stimulates TSG-6 involvement in this process. Elucidating the downstream regulatory network of TSG-6 would provide a clear theoretical basis for intervening in post-injury inflammatory responses (Pennati et al., 2025).

### 6.4 Development of disease-specific therapies

Developing disease-specific therapies using deer antler EVs. The core approach involves targeting and functionalizing these vesicles based on the unique pathological microenvironments of different tissue-damaging diseases, thereby achieving highly efficient and specific repair capabilities. Relevant studies indicate that preliminary data from mouse wound models indicate that encapsulating EVs-loaded pH/enzyme-responsive hydrogel dressings enables on-demand EVs release within acidic wound environments, thereby reducing inflammation and accelerating granulation tissue formation (Tan et al., 2024). Targeting core chronic wound pathologies—persistent inflammation and impaired tissue regeneration—this clinically applicable hydrogel dressing carrier offers significant implementation advantages. However, as an allogeneic biological carrier, deer antler EVs contain deer-specific proteins that may trigger immunogenic responses in humans, posing a core safety concern that limits their clinical application. This primarily manifests as dual activation of humoral and cellular immunity, which not only neutralizes the biological activity of the vesicles and weakens therapeutic efficacy but may also induce local inflammatory reactions or systemic allergic symptoms, severely compromising drug safety. Furthermore, functional modifications to the vesicles and the use of co-carriers may further alter their immunogenic characteristics. Therefore, immunogenicity assessments must cover the entire process—from preparation and modification to application—to mitigate potential risks. Concurrently, the clinical translation of this xenogeneic product faces multiple stringent regulatory barriers. Global regulatory bodies have yet to reach a unified consensus on their classification and evaluation standards. Differences exist in the regulatory positioning and core requirements of the FDA, EMA, and NMPA differ in their regulatory positioning and core requirements. Specific regulatory guidelines for xenogeneic EVs are lacking. Furthermore, the heterogeneity in deer antler sources—including species and rearing environments—makes it difficult to ensure batch-to-batch consistency. Relevant quality control indicators remain unstandardized, and long-term safety data are scarce. Compounded by ethical controversies surrounding xenogeneic biological products and variations in market access policies across regions, these factors collectively hinder the clinical translation process.

## 7 Conclusion

Deer antler EVs, leveraging the unique biological properties of their parental stem cells, emerge as highly promising novel nanocarriers in regenerative medicine. The specific bioactive components they carry can regulate core signaling pathways to directly promote stem cell proliferation and directed differentiation while simultaneously reshaping the immune microenvironment and modulating cytokine networks, demonstrating highly efficient tissue repair activity. Current Frontier approaches, such as engineered modifications and novel delivery system development, offer effective pathways to address bottlenecks like insufficient targeting and short *in vivo* retention time. However, challenges including immunogenicity risks, standardized preparation difficulties, inconsistent regulatory policies, and lack of long-term safety data continue to constrain clinical translation. Future efforts should focus on optimizing large-scale production techniques, elucidating molecular mechanisms, establishing unified quality evaluation systems, and advancing clinical trials to drive the practical application of deer antler extracellular vesicles in tissue repair.
